# 1993 Intercomparison of Photometric Units Maintained at NIST (USA) and PTB (Germany)

**DOI:** 10.6028/jres.100.016

**Published:** 1995

**Authors:** Yoshihiro Ohno, Georg Sauter

**Affiliations:** National Institute of Standards and Technology, Gaithersburg, MD 20899-0001; Physikalisch-Technische Bundesanstalt, Abteilung Optik, Photometrie, Postfach 33 45, 38023 Braunschweig, Germany

**Keywords:** candela, intercomparison, lumen, luminous intensity, luminous flux, photometer, responsivity, total flux, units

## Abstract

A bilateral intercomparison of photometric units between NIST, USA and PTB, Germany has been conducted to update the knowledge of the relationship between the photometric units disseminated in each country. The luminous intensity unit (cd) and the luminous flux unit (lm) maintained at both laboratories are compared by circulating transfer standard lamps. Also, the photometric responsivity *s*_v_ is compared by circulating a *V*(*λ*)-corrected detector with a built-in current-to-voltage converter. The results show that the difference of luminous intensity unit between NIST and PTB, (PTB-NIST)/NIST, is 0.2 % with a relative expanded uncertainty (coverage factor *k* = 2) of 0.24 %. The difference is reduced significantly from that at the 1985 CCPR intercomparison (0.9 %). The difference in luminous flux unit, (PTB – NIST)/NIST, is found to be 1.5 % with a relative expanded uncertainty (coverage factor *k* =2) of 0.15 %. The difference remained nearly the same as that at the 1985 intercomparison (1.6 %). These results agree with what is predicted from the history of maintaining the units at each laboratory.

## 1. Introduction

The last international intercomparison held by the CCPR (Comité Consultatif de Photométrie et Radiométric) in 1985 showed variations of ± 1 % in the units of both luminous intensity (candela, cd) and luminous flux (lumen, lm) maintained by the 14 and 10 participating laboratories, respectively [1]. At that time, the differences of the magnitude of units between NIST (National Institute of Standards and Technology, USA) and PTB (Physikalisch-Technische Bundesanstalt, Germany), (PTB–NIST)/NIST, were: 0.9 % for luminous intensity and 1.6 % for total luminous flux. Subsequently, BIPM (Bureau International des Poids et Mesures) and some of the participating laboratories changed their units, as reported by BIPM [2].

NIST and PTB have an agreement whereby each institution hosts staff members of the other every year and to collaborate on important scientific work in radiometry and photometry. This also provides a framework for bilateral intercomparisons. Since the CCPR intercomparison in 1985, only one bilateral intercomparison between NIST and PTB was conducted on the luminous flux unit in 1987 using a single incandescent lamp, OSRAM^1^ Wi5 (110 V/100 W frosted bulb). At that time the difference of the total luminous flux values measured by NIST and PTB, (NIST – PTB)PTB, was found to be 1.7 *%.*

To update the knowledge of the relationship of the photometric units, another bilateral intercomparison between NIST and PTB has been conducted. The luminous intensity units and the luminous flux units of both laboratories have been compared by circulating transfer lamps. The photometric responsivity *s*_v_ of a *V*(*λ*)^2^-corrected detector with a built-in current-to-voltage converter (called *photometer* in this paper) has also been compared.

The results of this intercomparison might also provide preliminary information to the CCPR in planning the next international intercomparison of photometric units [3]. CCPR established the Working Group on *V*(*λ*)-corrected Detectors in 1990, consisting of members from OMH (Országos Mérésügyi Hivatal, Hungary), NIST, NPL (National Physical Laboratory, UK), and PTB to prepare for the next intercomparison. This bilateral intercomparison has been performed partly based on the discussion at the ad hoc meeting of the Working Group held in May 1993 [4].

## 2. Realization and Maintenance of Photometric Units at NIST and PTB

At NIST, until recently, the luminous intensity unit (cd) was based on blackbody radiation [5]. There was a relative increase of the NIST unit by 0.35 % in 1990 due to the change of the international temperature scale. In 1992, the luminous intensity unit was realized at NIST based on the absolute responsivity of detectors, then based on a 100 % quantum-efficiency silicon detector [6], and now based on a cryogenic radiometer [7]. As a consequence of these more recent realizations of the unit, approximately 0.3 % relative change of the NIST luminous intensity unit is expected [6]. These changes in both 1990 and 1992 occurred in the direction in which the difference between NIST and PTB would be reduced.

**Fig. 1:**
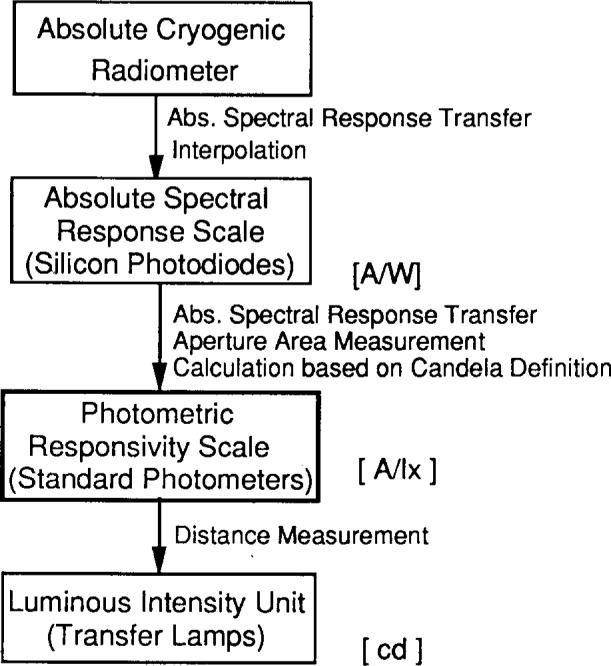
Realization and maintenance of the luminous intensity unit at NIST. (A/W: ampere per watt; A/Ix: ampere per lux.)

The new candela unit is realized and maintained on a group of eight photometers (referred to as the NIST *standard photometers).* Therefore, at NIST, photometric responsivity is the primary quantity used to derive photometric units. Illuminance is measured directly with the standard photometers, and the luminous intensity is inferred from the illuminance and the lamp-to-photometer distance. The realization and maintenance of the luminous intensity unit at NIST are shown in Fig. 1.

**Fig. 2:**
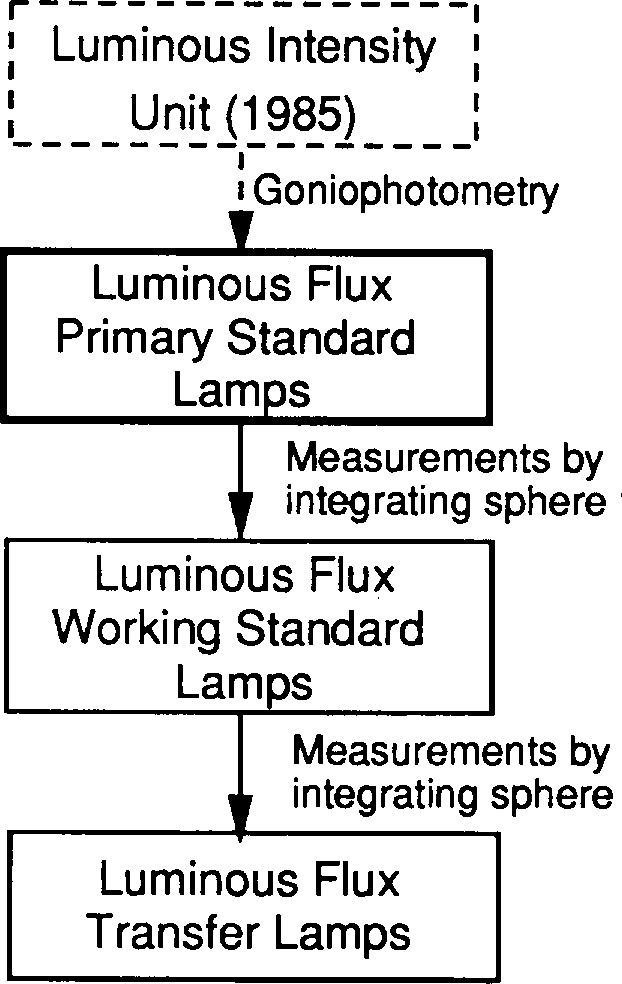
Realization and maintenance of the luminous flux unit at NIST.

The NIST luminous flux unit was last realized in 1985 by goniophotometric measurements [5], and maintained on a group of six incandescent lamps (referred to as the NIST *luminous flux primary standard lamps).* The unit is periodically transferred to batches of working standard lamps (referred to as NIST *luminous flux working standard lamps*) to be used for routine calibrations. The unit is currently independent of the NIST luminous intensity unit realized in 1992. The realization and maintenance of the luminous flux unit at NIST are shown in Fig. 2.

At PTB, the luminous intensity unit was first realized in 1980 based on radiometric power measurements with absolute radiometers, and the unit has been maintained on a batch of 23 incandescent lamps (Toshiba 5 A, 14 cd, 2042 K; referred to as the PTB *luminous intensity reference lamps*). Since then, the luminous intensity unit has been realized annually on two reference photometers based on the absolute radiometers. The unit realized on the reference photometers, however, has scattered around the unit on the reference lamps (within the uncertainty of realization), while the unit on the reference lamps has remained more stable. Therefore, the unit on the reference lamps has been maintained with no adjustment. This unit is transferred to batches of working standard lamps (referred to as PTB *luminous intensity working standards).*

**Fig. 3:**
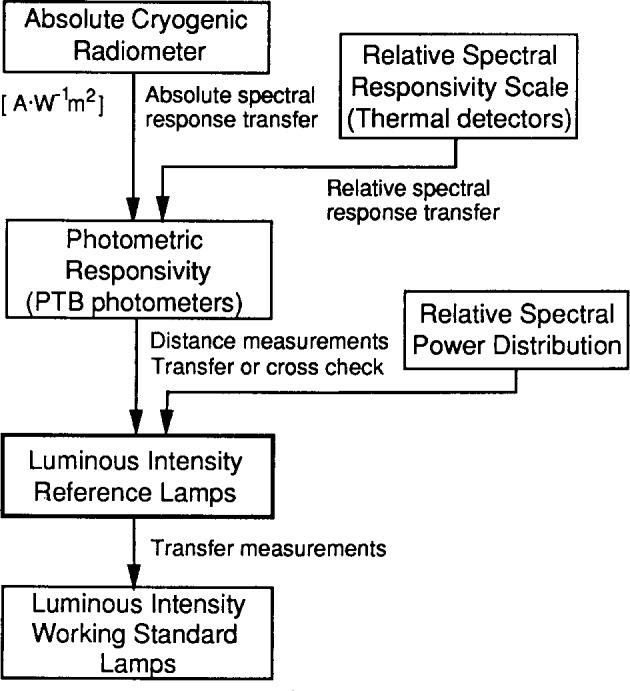
Realization and maintenance of the luminous intensity unit at PTB.

**Fig. 4:**
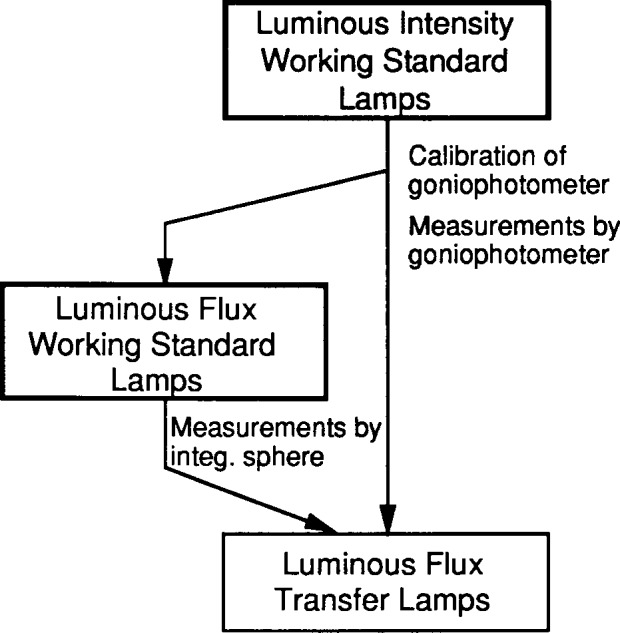
Realization and maintenance of the luminous flux unit at PTB.

The PTB luminous flux unit is derived from the luminous intensity unit by goniophotometric measurements and maintained by several batches of incandescent lamps (referred to as PTB *luminous flux working standard lamps).* All PTB photometric units have remained unchanged [8] since before the CCPR intercomparison in 1985. The realization and maintenance of the luminous intensity and luminous flux unit at PTB are shown in Figs. 3 and 4.

According to the results of the 1985 CCPR intercomparison and the history of maintaining the photometric units at both laboratories as described above, the relationship of the luminous intensity and luminous flux units between NIST and PTB is predicted as shown in Table 1. All the values are presented relative to the magnitude of world mean units as reported by BIPM [2].

## 3. Measurement Equations

Since the methods of photometric measurements used at NIST and PTB are in the main similar but differ in some details, the equations used in the calculation of the measurement results are given here systematically to clarify the concept and the calibration procedures at both laboratories. It should be noted here that a photometric responsivity is not regarded as a “photometric quantity” because all photometric quantities refer to properties of light sources according to CCPR [9]. The photometric responsivity, however, can be used to derive the photometric units, or can be used to maintain the illuminance units.

### 3.1 Equations for Luminous Intensity and Photometric Responsivity

With the maximum luminous efficacy, *K_m_ = 683* lm/W, as defined in 1979 by CGPM (Conférence Générate des Poids et Mesures) [10] and the spectral luminous efficiency function *V*(*λ*), which was adopted later by CCPR [9], luminous intensity *I* is calculated from the spectral radiant intensity *I*_e,λ_(λ)by

$$I=K_{m}\int_{\lambda }I_{e,\lambda }\left(\lambda \right)V\left(\lambda \right)d\lambda .$$ (1)

In this intercomparison, incandescent lamps are used as light sources. Since their relative spectral power distributions *S*(*λ,T*_d_) are similar to those of black body radiators, they are characterized by a distribution temperature *T*_d_ [11]. *I* is then given by

$$\begin{array}{l} I=K_{m}I_{e0}\int_{\lambda }S\left(\lambda ,T_{d}\right)V\left(\lambda \right)d\lambda \\ \text{with}\;I_{e,\lambda }\left(\lambda \right)=I_{e0}S\left(\lambda ,T_{d}\right), \end{array}$$ (2)

where *I*_e0_ is the spectral radiant intensity at wavelength λ_0_ = 555 nm. The spectral responsivity *s*(*λ*) of a photometer head is measured in radiometric units and is often given as the product of a normalization factor *s*_0_=*s*(*λ*_0_) and a function s_rel_ (*λ*) normalized at wavelength λ_0_ = 555 nm,

$$s\left(\lambda \right)=s_{0}s_{\text{rel}}\left(\lambda \right).$$ (3)

The normalization factor *s*_0_ is the absolute responsivity of the photometer head at wavelength *λ*_0_. The output signal *y* of the photometer, illuminated at a spectral irradiance *E*_e,_*_λ_*(*λ*) is given by

$$y=s_{0}\int_{\lambda }E_{e,\lambda }\left(\lambda \right)s_{\text{rel}}\left(\lambda \right)d\lambda .$$ (4)

According to the photometric inverse-square law *I*_e_ *= E_e_*·*l^2^*/*Ω*_0_, which defines the relationship between radiant intensity *I*_e_ and irradiance *E*_e_ (*Ω*_0_ is a solid angle equal to 1 steradian), the output signal *y* of the photometer, illuminated at a sufficiently large distance *l*, is expressed by

$$y=s_{0}I_{e0}\frac{\Omega_{0}}{l^{2}}\int_{\lambda }S\left(\lambda ,T_{d}\right)s_{\text{rel}}\left(\lambda \right)d\lambda .$$ (5)

The color correction function *ccf*(*T*_d_) is defined as

$$ccf\left(T_{d}\right)=\frac{\int_{\lambda }S\left(\lambda ,T_{d}\right)V\left(\lambda \right)d\lambda }{\int_{\lambda }S\left(\lambda ,T_{d}\right)s_{\text{rel}}\left(\lambda \right)d\lambda }$$ (6)

The basic equation for the realization of luminous intensity based on radiometric units –as done similarly by NIST and PTB–is derived from the combination of Eqs. (2), (5), and (6), and is given by

$$I=\frac{K_{m}}{s_{0}}\frac{l^{2}}{\Omega_{0}}y\;ccf\left(T_{d}\right).$$ (7)

In practice, a mismatch correction function ccf*(*T*d) normalized to unity at the distribution temperature *T*_d_ = 2856 K (CIE illuminant A) is often used, which is given by

$$ccf*\left(T_{d}\right)=\frac{ccf\left(T_{d}\right)}{ccf\left(T_{d}=2856\;K\right)}.$$ (8)

The related photometric responsivity $s_{v}^{*}$ is given by

$$s_{v}^{*}=\frac{s_{0}}{K_{m}ccf\left(T_{d}=2856\;K\right)}.$$ (9)

The photometric responsivity of a photometer changes with the temperature *T*_p_ of the photometer package by a temperature coefficient *c*_p_. A correction by a linear function has to be applied if the photometer is not temperature controlled. The correction is made by

$$s_{v}^{*}\left(T_{P}\right)=s_{v}^{*}+c_{P}\left(T_{P}-T_{P0}\right),$$ (10)

where *T*_p0_ is the temperature at which $s_{v}^{*}$ is determined. With the definitions of Eqs. (8) to (10), Eq. (7) can be rewritten as

$$I=\frac{y}{s_{v}^{*}\left(T_{P}\right)}\frac{l^{2}}{\Omega_{0}}cc\overset{*}{f}\left(T_{d}\right).$$ (11)

At PTB, the color correction function shown in Eq. (6) is replaced by a function *k*(*T*_d_), which is determined as a polynomial fit of *ccf*(*T*_d_) calculated at several (*n*) distribution temperatures in the range 2000 K⩽ *T_i_* ⩽2856 K with l⩽ i ⩽ *n* as given by

$$\begin{array}{c} \text{minimum}\;{\sum_{i=1}^{n}\left[k\left(T_{i}\right)-ccf\left(T_{i}\right)\right]}^{2}\\ \text{with}\;k\left(T_{d}\right)=\sum_{j=0}^{2}a_{j}T_{d}^{j}. \end{array}$$ (12)

Similar to Eqs. (8) to (11), normalization of Eq. (12) gives the mismatch correction function;

$$k*\left(T_{i}\right)=\frac{k\left(T_{i}\right)}{k\left(T_{d}=2856\;K\right)}$$ (13)

and the related photometric responsivity $s_{v}^{*}$;

$$s_{v}^{*}=\frac{s_{0}}{K_{m}k\left(T_{d}=2856\;K\right)}.$$ (14)

The corresponding equation to calculate the luminous intensity is

$$I_{v}=\frac{y}{s_{v}^{*}\left(T_{P}\right)}\frac{l^{2}}{\Omega_{0}}k*\left(T_{d}\right),$$ (15)

with the correction for the change of photometer package temperature *T*_p_ included.

### 3.2 Equations for Total Luminous Flux

The total luminous flux *Φ* of a lamp is fundamentally determined with a goniophotometer. Its value is calculated, starting with the definition of illuminance *E* =d*Φ/*d*A*, by the spatial integration of illuminance on an imaginary sphere with a total surface area *A*,

$$\Phi =\int_{\Lambda }E\;dA.$$ (16)

Using polar coordinates (*r*, *φ, ϑ*), with 0*⩽φ*⩽ 2π, 0*⩽ϑ*⩽, and dividing the surface area (radius *r*) into zones at angles *ϑ*, the equation can be rewritten as

$$\begin{array}{c} \Phi =2\pi r^{2}\int_{0}^{\pi }\bar{E}\left(\vartheta \right)\sin\left(\vartheta \right)d\vartheta \\ \text{with}\;\bar{E}\left(\vartheta \right)=\frac{1}{2\pi }\int_{0}^{2\pi }E\left(\vartheta ,\varphi \right)d\varphi . \end{array}$$ (17)

If *m* is the number of zones, the integration in *ϑ* is replaced by a summation with 1*⩽i⩽m*, Δ*ϑ* = π*/*(2m) (half-width of a zone) and *ϑ_i_*,=(2*i* − l) Δ*ϑ* (center angle of a zone). Then the equation is simplified to

$$\Phi =2\pi r^{2}\sum_{i=1}^{m}\bar{E}\left(\vartheta_{i}\right)\left(\cos\left(\vartheta_{i}-\Delta \vartheta \right)-\cos\left(\vartheta_{i}+\Delta \vartheta \right)\right).$$ (18)

The photometer in the goniophotometer is calibrated with luminous intensity working standards *I*_0_ at a distribution temperature *T*_0_ at a distance *l* with an output signal *y*_0_. At PTB, the total luminous flux *Φ* of a lamp under test, operated at distribution temperature *T*_d_, is calculated from the photocurrents $\bar{y}\left(\vartheta_{i}\right)$ averaged over zones,

$$\begin{array}{c} \Phi =2\pi \frac{I_{0}}{y_{0}}\frac{k*\left(T_{d}\right)}{k*\left(T_{0}\right)}{\left(\frac{r}{l}\right)}^{2}\sum_{i=1}^{m}\bar{y}\left(\vartheta_{i}\right)(\cos\left(\vartheta_{i}-\Delta \vartheta \right)\\ -\cos\left(\vartheta_{i}+\Delta \vartheta \right)). \end{array}$$ (19)

An integrating sphere is used to compare the values of total luminous flux *Φ* of a lamp under test — operated at distribution temperature *T*_d_ producing the output signal *y –*with the values of total luminous fluxes *Φ*_r,_*_i_* of (*n*) reference lamps – at distribution temperatures *T*_r,_*_i_* with 1⩽*i*⩽*n* and output signals *y*_r,_*_i_* If the holders of the lamps, sizes of the lamps, and/or the transparencies of the bulbs of the lamps are different, changes in self-absorption are corrected by the ratio of output signals y_ar,_*_i_* and y_a_ measured with the sphere illuminated by an auxiliary lamp and with a standard lamp or test lamp mounted in the sphere but not turned on. The following equation is used at PTB

$$\Phi =yk*\left(T_{d}\right)\frac{1}{n}\sum_{i=1}^{n}\left[\frac{\Phi_{r,i}}{y_{r,i}k*\left(T_{r,i}\right)}\frac{y_{\text{ar},i}}{y_{a}}\right].$$ (20)

In this equation, k*(*T*_d_) includes the mismatch of the photometer head modified by the relative spectral throughput of the integrating sphere. At NIST, the same equation is used with k*(*T*_d_) and k*(*T*_r,_*_i_*) replaced by ccf*(*T*_d_) and *ccf*(*T*_r,_*_i_*). As shown by Eqs. (10) and (11), corrections are applied for the changes of the photometer package temperature from a reference temperature.

## 4. Procedures of the Intercomparison

**Fig. 5:**
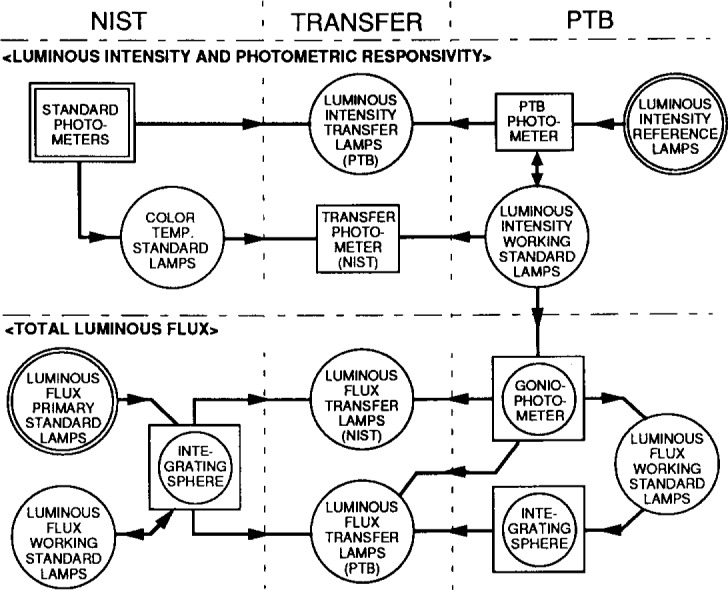
Structure of the intercomparison. Circles indicate lamps, squares indicate photometers. A circle in a square indicates instruments. Arrows mean the flow of calibration.

The measurements for this bilateral intercomparison were performed between April 1993 and December 1993. Measurements were carried out at both laboratories to compare the photometric values of 1) two *luminous intensity transfer lamps* prepared by PTB, 2) a *transfer photometer* prepared by NIST, and 3) four *luminous flux transfer lamps*, two of which were prepared by PTB and two by NIST. All the objects used in the intercomparison were hand-carried between NIST and PTB. The structure of the intercomparison is shown in Fig. 5.

At NIST, the standard photometers were used to measure the luminous intensity transfer lamps and the responsivity of the transfer photometer. A color temperature standard lamp (2856 K) was used in the responsivity measurement. The luminous flux transfer lamps were measured against the NIST luminous flux working standard lamps by a substitution method using a 2 m integrating sphere. The same procedures were used in the repeated measurements before and after the transportation of the lamps and the photometer.

At PTB, two luminous intensity transfer lamps were originally calibrated, as part of a group of six PTB luminous intensity working standard lamps against the PTB luminous intensity reference lamps. After return to PTB, the two transfer lamps were compared with the rest of the group of working standard lamps. These working standard lamps were also used to measure the photometric responsivity of the transfer photometer and to calibrate the PTB goniophotometer. Before transportation to NIST, the two luminous flux transfer lamps (PTB) were calibrated by a substitution method using an integrating sphere against a batch of PTB luminous flux working standard lamps, which were originally calibrated with the goniophotometer. After transportation to PTB, all the luminous flux transfer lamps and the luminous flux working standard lamps (PTB and NIST) were calibrated with the goniophotometer.

## 5. Luminous Intensity Comparison

Two luminous intensity transfer lamps were used to compare the unit of luminous intensity maintained at NIST and PTB. The lamps were operated at both laboratories under identical conditions and each lamp was calibrated twice at each laboratory in the order PTB→NIST→NIST→PTB. The measured values of luminous intensity are denoted as *I*_NIST_ and *I*_PTB_

### 5.1 Lamps Used

The two luminous intensity transfer lamps were of the type OSRAM Wi41/G, cemented in a special socket with a four-pole outlet for the electrical connections. They must be operated at constant dc current with a fixed polarity (negative potential connected to the center contact of the original lamp cap). The voltage across the lamp is measured to check the stability of the electric power consumed by the lamp. These lamps were used at PTB over a period of several years. The relative short-term stability of their luminous intensity was found to be better than 0.02 %. The relative aging during the burning of the lamps is estimated to be less than 0.03 %/h. The relative reproducibility due to alignment tolerances is found to be less than 0.05 %. The uncertainty of the intercomparison due to these factors in described in Sec. 5.4.

The socket is so constructed as to reduce the uncertainty due to alignment tolerances. To measure a number of lamps, only the lamp holder has to be aligned, which is done by an alignment jig (a socalled dummy lamp) constructed to give precise alignment using telescopes and a laser beam. The lamps can then be mounted into the holder without any further adjustment.

The lamp holder must be aligned so that 1) the center of the alignment jig is on the horizontal optical axis of the photometer, 2) the mirror surface of the alignment jig is perpendicular to the optical axis, 3) the upper plane of the lamp holder is horizontal as indicated by a level, 4) the distance measurement starts at a mark (hair cross) on the alignment jig.

### 5.2 Measurements at NIST

The luminous intensities of the transfer lamps were measured by using three of the NIST standard photometers. Measurements were first made in May 1993, when the lamps were brought to NIST, and repeated in October 1993, before the lamps were returned to PTB. The eight NIST reference photometers were recalibrated for self-consistency within the group between the two measurements in May and October. This means that each of the NIST photometers were recalibrated against the average of all the eight NIST photometers in order to reduce the errors due to long-term changes of individual photometers. The average change of the responsivity values of the three photometers used in the intercomparison was 0.08 %.

The measurements were made on the NIST photometry bench [6]. The lamp holder was fixed on a carriage of the bench, and aligned following the procedure specified by PTB using the alignment jig, a laser, and a telescope. The lamps were placed at approximately 3.5 m from the photometer and operated at a constant dc current with the polarity specified by PTB. The lamp current was automatically controlled, with a relative stability of ± 0.002 %. The voltage across the lamp during the measurement was recorded.

The luminous intensity *I*_NIST_ of the lamps was determined from the output voltage *y* of the photometer, the photometric responsivity $s_{v}^{*}$ of the photometer, and the measurement distance *l*. The temperature *T*_p_ of the standard photometer during calibration was monitored by a built-in temperature sensor, and the responsivity of the photometer was corrected using Eq. (10). The mismatch correction factors ccf*(*T*_d_) of the photometers shown in Eqs. (6) and (8) were applied for the difference between the color temperature for the calibration of the NIST photometers (2856 K) and that of the transfer lamps (2750 K). The luminous intensity *I*_NIST_ was calculated by Eq. (11).

### 5.3 Measurements at PTB

The mechanical alignment and the electrical operation of the lamps were carried out as stated in Sec. 5.1. The power supply was set with a resolution of better than 0.002 % of the current of the lamp, which was stabilized to better than ±0.001 *%* of the current during operation of the lamp. The relative expanded uncertainty^3^ of the electrical quantities is estimated to be 0.02 *%.*

In April 1993, the annual transfer of the luminous intensity unit to all working standards at PTB (including the two lamps transported to NIST) was carried out with two photometers at a distance of about 4.5 m. At the same time, the photometers were recalibrated and an aging of their mismatch correction functions was detected. The new function k*(*T_i_*) shown in Eq. (13) was not verified before transportation of the two luminous intensity lamps to NIST, so no correction was applied to the values first reported to NIST. Meanwhile, slightly different mismatch correction functions were obtained and the values of the luminous intensities of the two lamps (*T*_d_ = 2750 K) were increased by about 0.15 *%* of the original values.

After transportation back to PTB, the two lamps were recalibrated, but then four working standards (T_r_ = 2750 K) were used for the comparison with the PTB maintained unit. The transfer was carried out with the two photometers used in the annual transfer in April 1993. The distance to the lamps was adjusted to 3.5 m, the same as at NIST.

At PTB, photometer heads are temperature controlled and no correction for package temperature is applied. A substitution method was used for the two batches of lamps, both operated at the same distance from the photometers. The luminous intensity *I*_PTB_ of a lamp under test with distribution temperature *T*_d_, producing a photocurrent y is calculated from the luminous intensities *I*_r,_*_i_* and the related photocurrents *y*_r,_*_i_* for a batch of (*n*) reference lamps with the distribution temperature *T*_r,_*_i_* with 1 ⩽ *i* ⩽ *n* according to

$$I_{\text{PTB}}=yk*\left(T_{d}\right)\frac{1}{n}\sum_{i=1}^{n}\frac{I_{r,i}}{y_{r,i}k*\left(T_{r,i}\right)}.$$ (21)

The entire measurement procedure is run automatically by a computer.

### 5.4 Results

The results of luminous intensity measurements are summarized in Table 2. The average difference between the measured values of luminous intensity by NIST and PTB, (NIST – PTB)/PTB, was 0.18 % with a relative expanded uncertainty of 0.24 *%.* The uncertainty of this comparison is obtained as the quadrature sum of the statistical uncertainties of each laboratory’s luminous intensity measurements which should include the reproducibility of the lamps and the aging effect of the lamps. The statistical uncertainties at each laboratory were obtained as two times the standard deviation of the luminous intensity values of both lamps normalized by the average of each lamp.

Throughout these results, the maximum difference of luminous intensity of the same lamp measured twice at the same laboratory was 0.22 % of the measured values. The difference of NIST measurements in May and in October includes the slight changes of the responsivity values of the NIST standard photometers implemented by the self-consistency calibration as described in Sec. 5.2.

## 6. Photometric Responsivity Comparison

As described in Sec. 3.1, a photometer is characterized by the photometric responsivity $s_{v}^{*}\left(T_{p0}\right)$ and the mismatch correction function ccf*(*T*_d_) or k*(T_d_) determined at a certain temperature *T*_p0_ of the photometer package. At NIST, since the transfer photometer was not temperature controlled, the temperature coefficient *c*_p_ was additionally determined. The photometer was operated in both laboratories under identical photometric conditions and calibrated twice at each laboratory following the scheme NIST→PTB→PTB→NIST. The measured values of photometric responsivity are denoted as s*_NIST_ and s*_PTB_. Those of the mismatch correction functions are ccf*_NIST_(*T*_d_) and k*_PTB_(T_d_).

### 6.1 Photometer Used

The transfer photometer, prepared by NIST, is of the same type as the NIST reference photometers [7]. The photometer consists of an entrance aperture of 0.1 cm^2^ area, a *V*(*λ*)-correction filter (no opal glass), and a silicon photodiode with a sensitive area of 0.3 cm^2^. The reference surface for the distance measurement is the front surface of the aperture, which is 3 mm inside the front surface of the photometer. There is a built-in current-to-voltage converter with a gain setting from 10^4^ to 10^10^ (volt/ampere). The outside diameter of the photometer is 6.35 cm, and it operates on a dc potential of ± 15 V. It also has a built-in temperature sensor which measures the temperature of the photometer package. The temperature dependence of the photometer responsivity was found to be *c*_p_=−0.0176 mV·lx^−1^·K^−1^.

### 6.2 Measurements at NIST

The photometric responsivity of the transfer photometer was measured against two of the NIST standard photometers. Measurements were first made in May 1993 before the photometer was transported to PTB. Measurements were repeated in December 1993 when the photometer was transported back to NIST.

The measurements were made on the NIST photometry bench [6] using a color temperature standard lamp operated at *T*_d_ = 2856 K. Since all the NIST photometers are calibrated for 2856 K source, the spectral mismatch correction was not necessary. The distance between the photometer and the lamp was approximately 3.5 m. The ambient temperature was approximately 25 °C, and the temperature *T_p_* of the transfer photometer and *T*_p,_*_r_* of each standard photometer were measured and recorded at the same time as the output signal *y* was measured. The responsivity $s_{\text{NIST}}^{*}\left(T_{p}\right)$ of the photometer under test was determined at a range setting of 10^7^ from (1) the photometer temperature *T*_p_ measured, (2) the illuminance value determined by the standard photometers with photometric responsivity $s_{v,r}^{*}\left(T_{p,r}\right)$, and (3) the output signal *y*_r_ according to

$$s_{\text{NIST}}^{*}\left(T_{p}\right)=\frac{y}{y_{r}}s_{v,r}^{*}\left(T_{p,r}\right).$$ (22)

### 6.3 Measurements at PTB

At PTB, the transfer photometer was mounted in a temperature controlled housing at a constant temperature *T_p_* with |*T*_P_ *− T*_p0_|⩽0.5 K. The temperature coefficient *c*_p_ was not measured at PTB. The small deviation of the photometer temperature was taken into account to correct the responsivity using the temperature coefficient as given by NIST. All the measurements were carried out with the photometer aperture aligned normal and central to the optical axis of the photometer bench.

The mismatch correction function k*_PTB_(*T*_d_) of the transfer photometer was determined by substitution with a PTB photometer under the same illuminances by a standard lamp operated at varied color temperature. The mismatch correction function k*_r_(T_d_) of the PTB photometer was previously calibrated by spectral response measurements. k*_PTB_(*T*_d_) was determined from the photocurrents *y*(*T_i_*) of the transfer photometer and *y*_r_(*T_i_*) of the PTB photometer for the illuminances produced by the lamp operated at several (*n* = 4) distribution temperatures *T_i_*, as given by

$$\begin{array}{c} \text{minimum}\;{\sum_{i=1}^{n}\left[k_{\text{PTB}}\left(T_{i}\right)-\frac{y_{r}\left(T_{i}\right)}{y\left(T_{i}\right)}k_{r}^{*}\left(T_{i}\right)\right]}^{2}\\ \text{with}\;k_{\text{PTB}}\left(T_{i}\right)=\sum_{j=0}^{2}a_{j}T_{d}^{j}. \end{array}$$ (23)

The result *k*_PTB_(*T*_d_) was normalized to unity at the distribution temperature *T*_d_ = 2856 K to finally obtain the mismatch correction function k*_PTB_(*T*_d_).

The photometric responsivity s*_PTB_(*T*_p_) of the transfer photometer was measured by substitution with the PTB photometer. The photometric responsivity s*_v,r_ of the PTB photometer was first calibrated against four PTB luminous intensity working standard lamps. s*_PTB_(*T*_p_) was then determined, by comparison with the PTB photometer, from the photocurrent y, mismatch correction function k*_PTB_(*T*_d_), distribution temperature *T*_d_ of the lamp, the mismatch correction function k*_r_(*T*_d_) and photocurrent y_r_, of the PTB photometer

$$s_{\text{PTB}}^{*}\left(T_{P}\right)=\frac{k_{\text{PTB}}^{*}\left(T_{d}\right)}{k_{r}^{*}\left(T_{d}\right)}\frac{y}{y_{r}}s_{v,r}^{*}.$$ (24)

### 6.4 Results

Table 3 shows the results of the determination of the photometric responsivity $s_{\text{PTB}}^{*}\left(T_{d}\right)$ and $s_{\text{NIST}}^{*}\left(T_{d}\right)$ of the transfer photometer. Since the temperature of the transfer photometer was different when measured at NIST and at PTB, the values are corrected to the same temperature (297.0 K). The difference of the photometric responsivity measured by NIST and by PTB, (NIST – PTB)/PTB, was − 0.22 %, with a relative expanded uncertainty of 0.14 %. The uncertainty of this comparison is obtained as the quadrature sum of the statistical uncertainties of each laboratory’s measurements, which were obtained as two times the standard deviation of the photometric responsivity values measured.

**Fig. 6:**
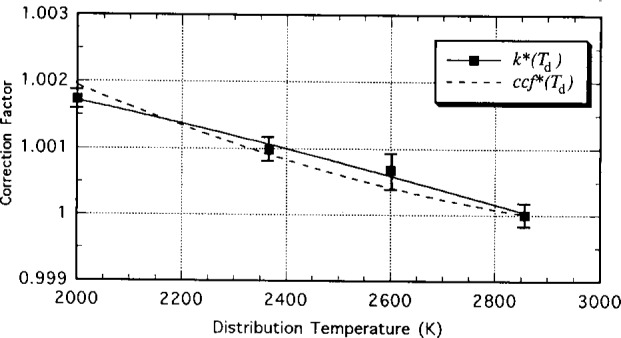
Mismatch correction values k*(*T_i_*) and the fitted function k*(*T*_d_) determined at PTB (solid curve), and the mismatch correction function ccf*(*T*_d_) measured at NIST (dashed curve). The uncertainty bars show 2 standard deviations of the PTB measurements.

Table 4 shows the results of the mismatch correction function of the NIST transfer photometer calculated from four distribution temperatures. $k_{\text{PTB}}^{*}\left(T_{d}\right)$ is the value measured by PTB and $k_{\text{NIST}}^{*}\left(T_{d}\right)$ by NIST. The coefficients *a*_r,_*_j_* of the fitted second order polynomial are also shown. Fig. 6 shows the mismatch correction function curves obtained by PTB and by NIST. The mismatch correction factors of the transfer photometer in a range 2000 K to 3000 K measured by NIST and by PTB agreed within ± 0.0003.

## 7. Total Luminous Flux Comparison

Four luminous flux transfer lamps –two from NIST and two from PTB–were used to compare the unit of luminous flux maintained at NIST and PTB. The lamps were operated at both laboratories under identical conditions. Each lamp was calibrated twice at each laboratory following the schemes NIST→PTB→PTB→NIST for the NIST luminous flux transfer lamps, and PTB→NIST→NIST→PTB for the PTB luminous flux transfer lamps. The measured values of total luminous flux are denoted as *Φ*_NIST_ and *Φ*_PTB_.

### 7.1 Lamps Used

Two OSRAM Wi4 (24 V/100 W frosted bulb) lamps were prepared by PTB, and two OSRAM 24 V/40 W opal bulb lamps were prepared by NIST. These transfer lamps were operated at constant dc current with specified polarity and operated in the cap-up position. The lamps prepared by NIST were seasoned for at least 50 h on a dc power at approximately the same current as used in the measurement. The color temperature of the NIST lamps (2750 K) was determined using a spectroradiome-ter. The lamps prepared by PTB belong to a group of working standards used for several international and bilateral intercomparisons in European countries. Their stability (short-term) and reproducibility over several years is known to be better than 0.1 *%.* The distribution temperature (2830 K) was determined from the spectral distribution.

### 7.2 Measurements at NIST

The total luminous fluxes of the transfer lamps were measured using a 2 m integrating sphere [7] against the NIST luminous flux working standard lamps. All transfer lamps were operated, in the base-up position, at dc constant current with the specified polarity. The lamp voltage during the measurement was recorded. The integrating sphere is equipped with a *V*(*λ*)-corrected detector of a design similar to the NIST standard photometers. The detector has an opal diffuser at the entrance for cosine correction. The detector has a built-in temperature sensor, and the output reading is corrected for the differences in temperatures during measurements as shown by Eq. (10). The integrating sphere is equipped with an auxiliary lamp on the sphere wall, and the self-absorption effects are measured for each lamp including lamp holders, and corrections are made to the results. The relative spectral responsivity of the detector and the relative spectral throughput of the integrating sphere were measured. The mismatch correction factors as given by. Eqs. (6) and (8) were calculated and applied for the differences in color temperature of the lamps measured. The total luminous flux values were calculated according to Eq. (20) with k*(*T*_d_) replaced by ccf*(*T*_d_).

### 7.3 Measurements at PTB

In April 1993, when the two luminous flux transfer lamps were transported to NIST, the PTB goniophotometer had not yet finished being upgraded. As a result, PTB calibrated the luminous flux of the lamps using a 1.5 m integrating sphere. The lamps were compared with luminous flux working standard lamps, which had been calibrated with the PTB goniophotometer one year before. The luminous flux values were calculated according to Eq. (20).

When the luminous flux transfer lamps were transported back to PTB, the upgrade of the PTB goniophotometer was complete, and all the lamps were calibrated with the goniophotometer. The goniophotometer was calibrated against the PTB luminous intensity working standard lamps. These are the same lamps used to calibrate the luminous intensity transfer lamps and the photometric responsivity of the NIST photometer.

The total luminous flux values of the transfer lamps were calculated automatically using the goniophotometer program according to Eq. (18). The radius of the goniophotometer is ~ 2.5 m and the surface of the imaginary sphere is divided into ~70 zones. Corrections for photometer spectral mismatch, stray light and drift of the NIST lamps during the measurement time (about 20 min for one run) are applied to the values. The drift is measured with a monitor photometer moving continuously only at the equatorial zone.

### 7.4 Results

The results of the total luminous flux measurements are summarized in Table 5. The average difference between the measured values of luminous intensity by NIST and by PTB, (NIST − PTB)/PTB, was 1.53 %, with a relative expanded uncertainty of ±0.15 %. The uncertainty of this comparison is evaluated in the same way as that of the luminous intensity comparison was.

## 8. Conclusion

The bilateral intercomparison of photometric units maintained at NIST and at PTB was conducted within a 1-year period. Two luminous intensity transfer lamps, one *V*(λ)-corrected transfer photometer and four luminous flux transfer lamps were calibrated at NIST and PTB against the maintained units of luminous intensity and luminous flux. All the measurements were repeated by the two laboratories in such a way that four values were always obtained: the first and the fourth at one laboratory before and after the transportation, and the second and third at the other laboratory with approximately 3 months in between.

The luminous intensity values measured by NIST were found to be 0.18 % (of the measured values) larger than the values measured by PTB with a relative expanded uncertainty of 0.24 %. The photometric responsivity value of the transfer photometer for CIE illuminant A measured by NIST was found to be 0.22 % (of the measured value) smaller than the value measured by PTB with a relative expanded uncertainty of 0.15 %. This means that the magnitude of the NIST candela is 0.2 %± 0.24 % (of the unit) smaller than that of the PTB candela. The difference is well within the relative expanded uncertainty of calibration at each laboratory (0.5 % at NIST [7] and 0.4 % at PTB [12]), and is significantly reduced from a value of 0.9 % obtained in 1985. The comparisons using the lamps and the photometer gave almost equivalent results. The mismatch correction factors of the transfer photometer measured in a range 2000 K-3000 K by NIST and by PTB agreed within ±0.0003.

The total luminous flux values measured by NIST were found to be 1.53 % (of the measured values) larger than the values measured by PTB with a relative expanded uncertainty of 0.15 %. In other words, the NIST lumen is 1.53 %±0.15 % (of the unit) smaller than the PTB lumen. The result indicates, however, that the luminous flux units maintained at both laboratories have not changed significantly since 1985. The difference in luminous flux unit is significant compared with the relative expanded uncertainty of calibration at each laboratory (1.0 % at NIST [7] and 0.6 % at PTB [12]). This difference should be studied. At NIST, a new realization of luminous flux unit is in progress.

Throughout the results of this intercomparison, the reproducibility of lamps after a round trip to and from NIST and PTB showed satisfactory results with a maximum change of 0.11 % of the luminous intensity and 0.13 % of the total luminous flux, which were comparable with the change of 0.09 % of the responsivity of the photometer. The reproducibility might be degraded if the objects are not hand-carried. It is also noted that the results for both luminous intensity and luminous flux units agreed with what had been expected from the history of maintaining the units at each laboratory.

## Figures and Tables

**Table 1:** Relationship of NIST and PTB units of luminous intensity (candela, cd) and luminous flux (lumen, lm) since 1985 CCPR intercomparison, relative to the world mean of 1985

Units	Participant	1985 CCPR intercomp.	1987 Bilateral intercomp.	1988 BIPM report	1990 New Int’n Temp. Scale	1992 NIST New unit	1993 Predicted
Candela	BIPM	1.0100		1.000			1.000
	PTB	1.0032		1.003			1.003
	NIST	0.9942		0.994	0.9977	1.0014	1.001
	PTB–NIST	0.0090		0.009	0.0055	0.0018	0.002
Lumen	BIPM	0.9930		1.000			1.000
	PTB	1.0056		1.006			1.006
	NIST	0.9899		0.990			0.990
	PTB–NIST	0.0157	0.017	0.016			0.016

**Table 2:** Summarized results for PTB luminous intensity transfer lamps measured at NIST and PTB with average differences between the values

Lamp no. lamp current dist. temp.	Date 1993	Lamp voltage V		Luminous intensity *I* cd		Difference	Burning time min

		NIST	PTB	NIST	PTB	(NIST–PTB)/PTB	
260S	March		27.847		196.22		40
5.500 A	May	27.851		196.65			60
2740 K	October	27.850		196.39			60
	October		27.845		196.06		40

	Average	27.851	27.846	196.52	196.14	0.19 %	Total 200
	Δ^a^	0.00%	0.01 %	0.13 %	0.08 %		

264S	March		27.953		198.64		80
5.500 A	May	27.956		199.09			60
2750 K	October	27.957		198.64			60
	October		27.950		198.43		40

	Average	27.957	27.952	198.86	198.54	0.16 %	Total 240
	Δ	0.00%	0.01 %	0.23 %	0.11 %		

*U*_lab_^b^				0.21 %	0.10 %		

Difference of luminous intensity values measured by NIST and by PTB: + 0.18 % ± 0.24 *%*(*U*)							

a Δ is the difference of the two measurements of each lamp at each laboratory, [*I*(first)-*I*(second)]/*I*(first).

b U_lab_ is two times the standard deviation of luminous intensity values of each laboratory normalized by the average of each lamp. *U* is the quadrature sum of *U*_lab_(NIST) and *U*_lab_(PTB).

**Table 3:** Determination of the photometric responsivity of the NIST transfer photometer compared to that of a PTB photometer

Date of measurement 1993	Pack. temp. *T*_p_ K	Photometric responsivity s^*^(*T*_p_) mV/lx		Corrected responsivity s^*^(297.0) mV/lx		Difference

		NIST	PTB	NIST	PTB	(NIST-PTB)/PTB
April	297.0	23.120		23.120		
August	297.5		23.174		23.183	
October	297.1		23.176		23.178	
December	297.4	23.135		23.142		

Average	297.3			23.131	23.181	−0.22%
Δ^a^				0.09%	0.02 %	

*U*_lab_^b^				0.13 %	0.03 %	

Difference between photometric responsivity values measured by NIST and PTB: −0.22 %±0.14 %(U)						

a Δ is the difference of the two measurements at each laboratory, [s^*^(first)-s^*^(second)]/s^*^(first).

b *U*_lab_ is two times the standard deviation of the responsivity values at each laboratory. *U* is the quadrature sum of *U*_lab_(NIST) and *U*_lab_(PTB).

**Table 4:** Determination of the mismatch correction function of the NIST photometer by a parabolic fitting from values measured at four different distribution temperatures

	PTB	NIST

*T*_d_	k^*^_PTB_(*T*_d_)	k^*^_NIST_(*T*_d_)
2000	1.00173	1.00194
2366	1.00098	1.00090
2600	1.00068	1.00042
2856	1.00000	1.00000

*a*_0_	1.00313	1.0129
*a*_1_	1.4525 × 10^−7^	−7.7218 × 10^−6^
*a*_2_	−4.341 × 10^−10^	1.1248 × 10^−9^

**Table 5:** Summarized results for PTB and NIST luminous flux transfer lamps measured at NIST and PTB including relative differences between the values and the related statistical uncertainties

Lamp no. lamp current dist. temp.	Date 1993	Lamp voltage V		Luminous flux *Φ*		Difference	Burning time min

		NIST	PTB	NIST	PTB	(NIST−PTB)/PTB	
PTB9	April		21.497		1231.0		30
3.800 A	May	21.511		1249.5			45
2828 K	October	21.510		1250.2			45
	November		21.501		1232.6		60

	Average	21.511	21.499	1249.9	1231.8	1.47 %	Total 180
	Δ^a^	0.01 %	0.02 %	−0.06 %	−0.13 %		

PTB10	April		21.863		1287.0		30
3.800 A	May	21.877		1304.4			45
2844 K	October	21.870		1306.2			45
	November		21.867		1287.9		60

	Average	21.874	21.865	1305.3	1287.5	1.38 %	Total 180
	Δ	0.03 %	0.02%	−0.14 %	−0.07%		

NIST1205	October	24.080		489.8			45
1.660 A	November		24.082		481.39		60
2760 K	November		24.084		482.14		60
	December	24.097		489.8			20

	Average	24.089	24.083	489.8	481.77	1.67 %	Total 185
	Δ	0.07 %	0.01 %	0.00%	−0.15 %		

NIST1206	October	24.240		510.9			45
1.660 A	November		24.242		502.87		60
2776 K	November		24.243		502.75		60
	December	24.245		510.6			20

	Average	24.243	24.243	510.75	502.81	1.58 %	Total 185
	Δ	0.02 %	0.00%	0.06%	0.02 %		

*U*_lab_^b^				0.09%	0.12 %		

Difference between luminous flux values measured by NIST and PTB: +1.53 %±0.15 *%*(*U*)							

a Δ is the difference of the two measurements of each lamp at each laboratory, [*Φ*(first)−<*Φ*(second)]/*Φ*(first).

b *U*_lab_ is two times the standard deviation of luminous flux values of each laboratory normalized by the average of each lamp. *U* is the quadrature sum of *U*_lab_(NIST) and *U*_lab_(PTB).

## Footnotes

1 Certain commercial equipment, instruments, or materials are identified in this paper to foster understanding. Such identification does not imply recommendation or endorsement by the National Institute of Standards and Technology, nor does it imply that the materials or equipment identified are necessarily the best available for the purpose.

2 *V*(*λ*) is the spectral luminous efficiency of human vision, standardized by CIE (International Commission on Illumination) in 1924.

3 Throughout this paper, all uncertainties are given as relative expanded uncertainties with a coverage factor *k* = 2 and thus a 2 standard deviation estimate.
